# In Vitro Antithrombotic, Hematological Toxicity, and Inhibitor Studies of Protocatechuic, Isovanillic, and *p*-Hydroxybenzoic Acids from *Maclura tricuspidata* (Carr.) Bur

**DOI:** 10.3390/molecules27113496

**Published:** 2022-05-29

**Authors:** Jun-Hui Choi, Seung Kim

**Affiliations:** Department of Health Functional Food, Gwangju University, Gwangju 61743, Korea; sekai0572@naver.com

**Keywords:** protocatechuic acid, isovanillic acid, *p*-Hydroxybenzoic acid, antithrombosis, anticoagulation, inhibitor

## Abstract

In blood coagulation, circulating platelets and coagulation factors are crucial for the primary process because thrombi are generated by fibrin clotting with fibrinogen, thrombin, FXIIIa, and platelet activation. Therefore, strategies to reduce the activity of key coagulation factors, or interfere with their functions and delay the activation of platelets can be used as important tools to suppress excessive blood clot formation and platelet hyperactivation. This study examined the antithrombotic activity and hematological toxicity of PA, IVA, and 4-HA isolated from *M. tricuspidata* (Carr.) Bur in several in vitro experiments and inhibitor assays. We found that PA, IVA, and 4-HA attenuated the formation of fibrin polymers/clots and degraded the blood clots. These compounds inhibited the activities of procoagulant proteases and fibrinoligase, and prolonged the coagulation time. There was a significant reduction in platelet function and ATP or serotonin levels in thrombin-activated platelets. An inhibitor study showed that PA exhibited a mixed inhibition type for thrombin, an uncompetitive inhibition type for FXa, and a non-competitive inhibition type for FXIIIa and IVA, while 4-HA exhibited an uncompetitive inhibition type for thrombin and non-competitive inhibition type for FXa and FXIIIa. These three compounds (up to 50 μg/mL) were not toxic to blood cells.

## 1. Introduction

Blood coagulation process is complex process involving the sequential activation of various factors. Most blood coagulation factors exist in an inactive precursor protein state in the plasma, and participate in blood coagulation after sequential activation [1]. The blood coagulation mechanism following sequential activation of blood coagulation factors includes the intrinsic, extrinsic, and common pathways [2]. When the blood vessel wall is damaged, the tissue factor, blood coagulation factor III, is expressed in the vascular endothelial cells. Tissue factors rapidly bind to the blood coagulation factor VII to initiate the sequential activation of other blood coagulation factors. Eventually, because of this activation, fibrinogen is converted into fibrin, forming fibrin clots. Fibrinogen, containing two sets of three polypeptide chains (Aα, Bβ, and γ) is a water-soluble plasma protein that is made up of fibrin, a major structural element of thrombus, obtained via proteolysis of thrombin [3]. The formation of fibrin clots is closely correlated to platelets and activated factor XIII [4,5]. Excessive thrombus formation or imbalance of these systems causes cardiovascular diseases, such as stroke, myocardial infarction, deep-vein thrombosis, and pulmonary embolism [6].

We recently studied the anti-obesity and antithrombotic effects of the edible and medicinal plant, *M. tricuspidata* (Carr.) Bur, and isolated several flavonoids and polyphenols from it [7,8]. *Maclura tricuspidata* (Carr.) Bur belongs to the family Moraceae and is mainly distributed in Korea, Japan, and China. Its fruit is used for the production of drinks and food items, while its roots and bark are used as medicinal materials [7]. Different parts of *M. tricuspidata* (Carr.) Bur exhibit antioxidant, antimicrobial, anticancer, and antihypertensive effects [9]. In particular, several studies have demonstrated its protective effects on blood circulation and cardiovascular health, such as improving insulin resistance, plasma triglyceride levels, and platelet function, while preventing obesity and diabetes [10,11]. We observed the antithrombogenic, anticoagulation effects, and inhibitory effects of protocatechuic acid (PA), isovanillic acid (IVA), and 4-hydroxybenzoic acid (4-HA). Several studies have investigated the anti-platelet aggregation and antithrombotic activities of PA, IVA, and 4-HA [12,13,14,15,16,17], but there are no studies on the effect of these compounds on cell viability of blood cells and their inhibitory effects on fibrin clot formation, enzymatic activities of procoagulant proteases or fibrinoligases, and plasma recalcification. Therefore, this study was designed to investigate the inhibitory effects of these compounds against thrombogenesis and coagulation of blood and determine their potential to be used as novel antithrombotic and anticoagulation agents. The effects of these compounds on fibrin clot formation and procoagulant protease activity were investigated. Anti-platelet and anticoagulation actions were evaluated by measuring the epinephrine/collagen-stimulated platelet activation or coagulation time. The viabilities of blood cells, including platelets, leukocytes, and erythrocytes, were investigated.

## 2. Results

### 2.1. Effects of PA, IVA, and 4-HA on the Viability of Blood Cells

**Figure 1:**
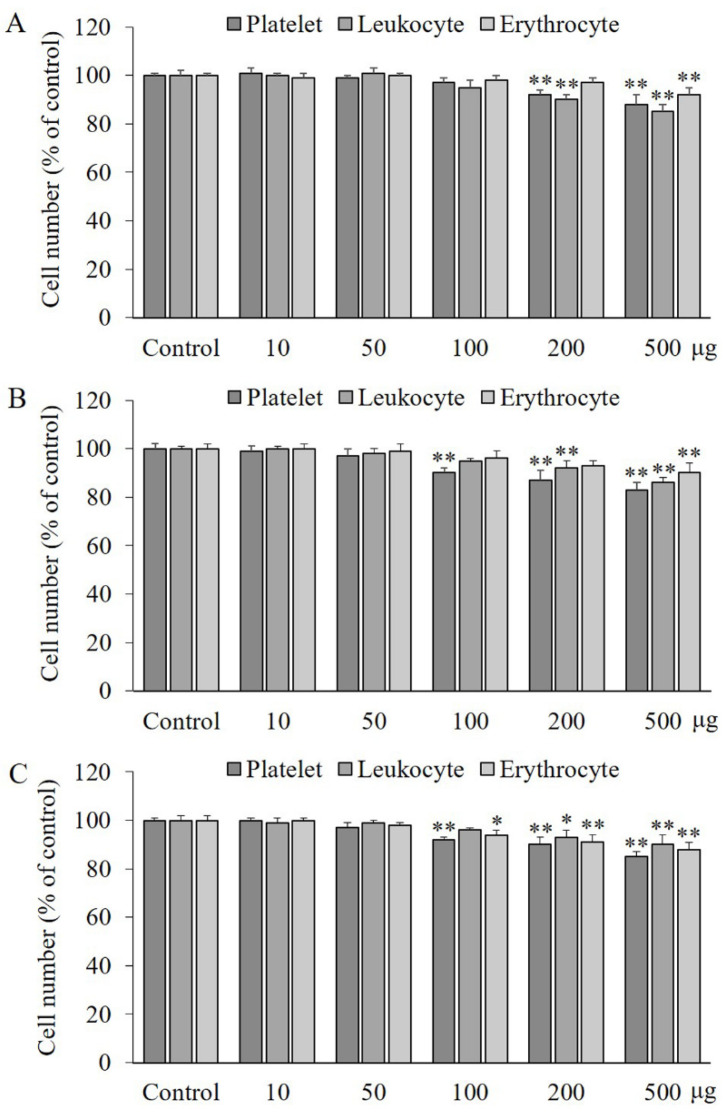
Blood cytotoxic effect of PA (**A**), IVA (**B**), and 4-HA (**C**). Aliquots of washed platelets (10^3^/mL), erythrocytes (10^6^/mL), and leukocytes (10^3^/mL) were treated with or without the compounds for 10 min and cell number was determined by hemocytometer counts. Each value is the mean ± SD of triplicate measurements. * *p* < 0.05 and ** *p* < 0.01, compared to each control groups. 10–500, 10–100 μg of each compound treated.

PA or IVA treatment up to 200 μg/mL did not show any effect on the number of erythrocytes, while treatment with 10–100 μg/mL of PA or 10–50 μg/mL of IVA or 4-HA did not cause any effect on the platelets and leukocyte numbers, compared to the control group, as shown in Figure 1A–C. However, treatment with the three compounds at a concentration of 500 μg/mL significantly decreased (*p* < 0.01) the number of all blood cells.

### 2.2. PA, IVA, and 4-HA Attenuate Fibrin/Blood Clot Formation

**Figure 2:**
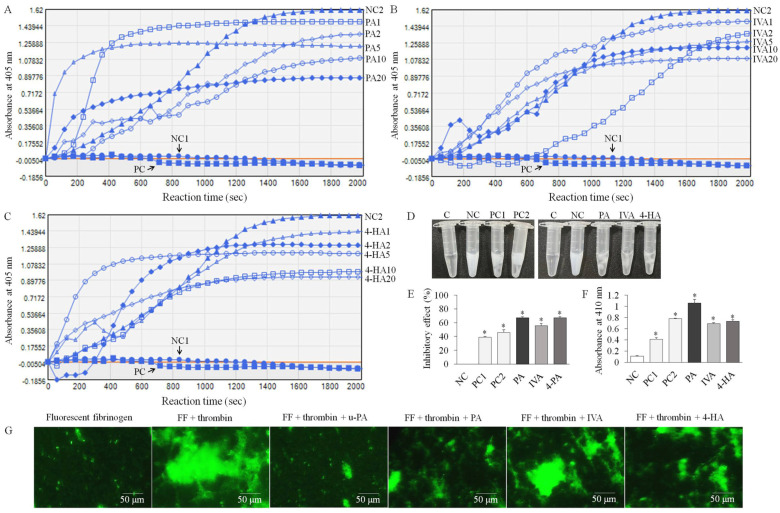
In vitro antithrombotic effects of PA, IVA, and 4-HA. (**A**–**C**) Turbidity assay. (**D**,**E**) Fibrin clot assay. (**F**) In vitro antithrombotic activity assay using blood clot. (**G**) Fibrin clot assay using a fluorescence microscope. Each value is the mean ± SD of triplicate measurements. * *p* < 0.01, compared to the non-treated control (NC) group. NC1, fibrinogen only; NC2, fibrinogen treated with thrombin; PC, positive control group (10 IU u-PA); PA1–20, 1–20 μg of PA treated; IVA1–20, 1–20 μg of IVA treated; 4-HA1–20, 1–20 μg of 4-HA treated; PC1–2, 10–20 IU u-PA; FF, fluorescent fibrinogen.

As shown in Figure 2, changes in the absorbance of fibrin polymer turbidity were tracked over time for 2000 s at 405 nm in each group. Fibrin polymers and clots were formed by the reaction of fibrinogen, thrombin, CaCl_2_, and different concentrations of compounds (1, 2, 5, 10, and 20 μg), and the absorbance of the clots increased gradually in the negative control group (NC) 2 group compared to the NC1 group treated with only fibrinogen and the PC group treated with fibrinogen, thrombin, and urokinase-type plasminogen activator (u-PA). At certain concentrations (1, 5, or 20 μg) of the compounds, the polymers were formed more rapidly, but eventually after the reaction time, fibrin polymer/clot formation was dose-dependently inhibited by PA, IVA, and 4-HA treatment (Figure 2A–C). In the fibrin clot assay, clots were strongly formed by thrombin activation in the NC group compared to the NC group containing only fibrinogen (Figure 2D). PA, IVA, 4-HA, and u-PA inhibited fibrin clot formation by 66.9 ± 3.3, 55.6 ± 1.7, and 67.1 ± 1.3% at a concentration of 100 μg and 39–46% at 10–20 IU, respectively (Figure 2E). In in vitro antithrombotic activity assay, PA, IVA, 4-HA, and u-PA significantly degraded (*p* < 0.01) the blood clot formation, compared to the NC group treated with saline (Figure 2F). The inhibitory action of the compounds on the fibrin network structure was visualized using Alexa Fluor 488-labeled fibrinogen and fluorescent microscopy. Figure 2G shows the intensive fibrin clot network treated with fluorescent fibrinogen and thrombin, whereas in the experimental groups treated with PA, IVA, 4-HA, or u-PA, morphological changes in the network and reduced fibrin clot density were observed.

### 2.3. PA, IVA, and 4-HA Decrease the Enzymatic Activities of Procoagulant Proteinases

**Figure 3:**
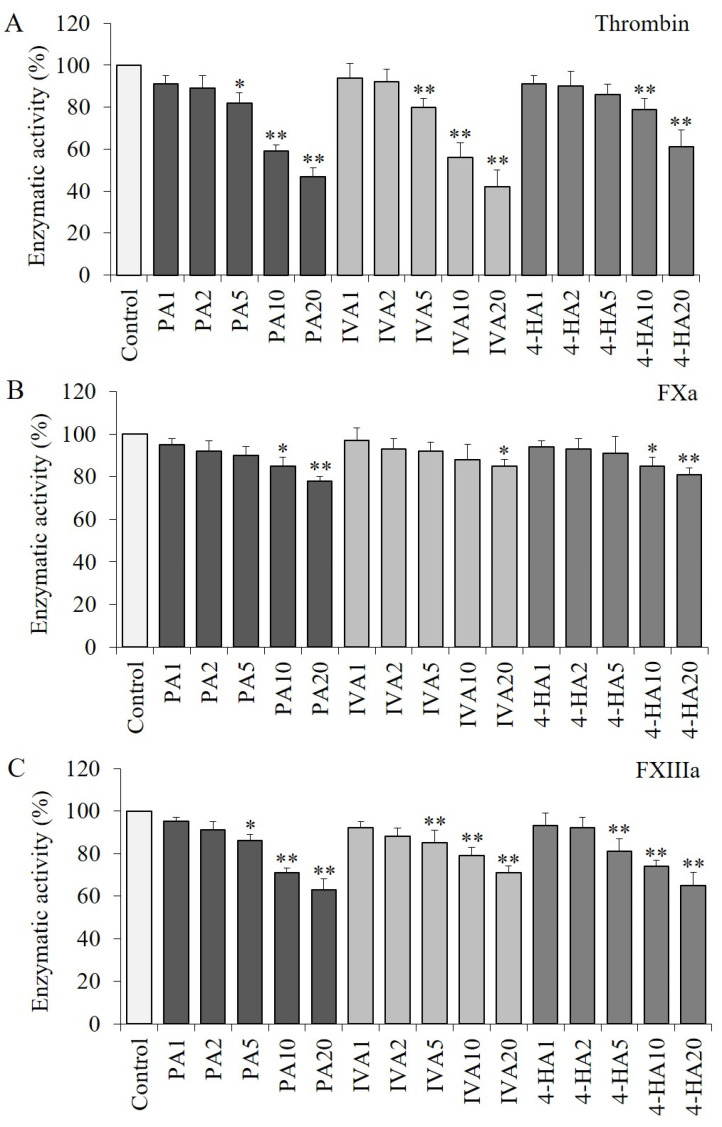
Effects of PA, IVA, and 4-HA on thrombin (**A**), FXa (**B**), and FXIIIa (**C**) activity. Each value is the mean ± SD of triplicate measurements. * *p* < 0.05 and ** *p* < 0.01, compared to each control groups. Control, enzyme only; PA1–20, 1–20 μg of PA treated; IVA1–20, 1–20 μg of IVA treated; 4-HA1–20, 1–20 μg of 4-HA treated.

The activity of thrombin was significantly decreased (*p* < 0.01) by PA, IVA, or 4-HA at the highest concentration of each compound (Figure 3A). Likewise, significant reductions (*p* < 0.05) were confirmed in the PA-, IVA-, or 4-HA-treated activated factor X (FXa) or activated factor XIII (FXIIIa) groups compared to the control group without any compound treatment (Figure 3B,C).

**Figure 4:**
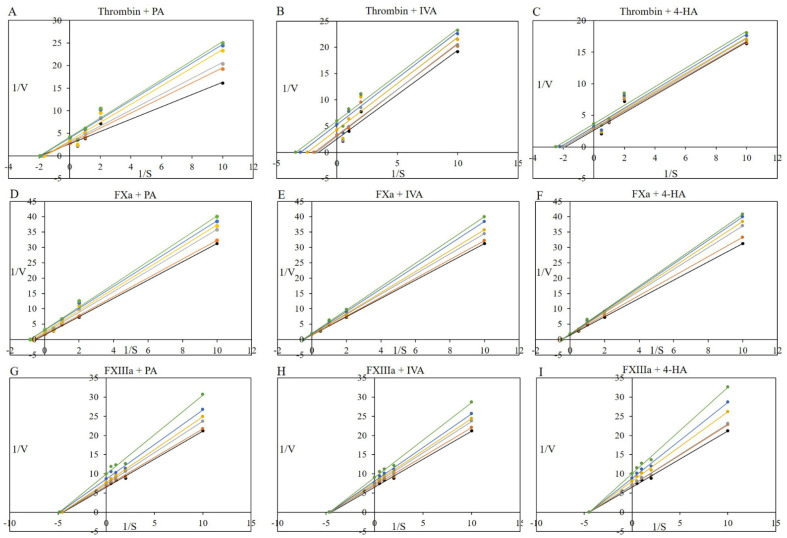
Kinetic and inhibitor studies for thrombin, FXa, and FXIIIa. Lineweaver—Burk plots were used for the inhibition of target enzymes. The plots are expressed as 1/velocity (1/V) versus 1/substrate (1/[S]) with or without each compound (non-treated (black circle), 0.065 (orange circle, 1 μg), 0.13 (silver circle, 2 μg), 0.325 (yellow circle, 5 μg), 0.65 (blue circle, 10 μg), and 1.3 mM (green circle, 20 μg)) as an inhibitor against thrombin (**A**–**C**), FXa (**D**–**F**), and FXIIIa (**G**–**I**).

We further determined the kinetic and inhibitory constants of PA, IVA, and 4-HA against procoagulant proteases (thrombin and FXa) and fibrinoligase using the specific substrates (HD-Phe-Pip-Arg-*p*-nitroanilide, *N*-benzoyl-Ile-Glu-Gly-Arg-*p*-nitroanilide, and casein/fibrinogen-HRP) as shown in Figure 4. As shown in Table 1, the *K*_m_ value of thrombin was found to be 0.566 ± 0.025 mM, and this constant of thrombin was reduced by 0.89–0.91-fold (PA), 0.52–0.93-fold (IVA), and 0.71–0.86-fold (4-HA). The *V*_max_ value of thrombin decreased by 0.65–0.92-fold (PA), 0.44–0.86-fold (IVA), and 0.72–0.92-fold (4-HA), compared to the non-treated control group (0.381 ± 0.011 mU/min). Treatment with PA, IVA, and 4-HA reduced the catalytic rate constant (*K*_cat_) values of thrombin by 0.63–0.93-fold, 0.43–0.86-fold, and 0.72–0.92-fold, respectively, compared to the non-treated control group (109.87 ± 3.16 s^−1^), and there was a significant reduction in the mean the catalyst efficiency (*K*_cat_/*K*_m_) values of thrombin with all treatments, except 4-HA treatment, compared to the non-treated control group (196.33 ± 12.61 mM^−1^s^−1^). The inhibition constant using *K*_m_ (*K*_ik_)/the inhibition constant using *V*_max_ (*K*_iv_) ratio values for thrombin inhibition were determined to be 125.4 by PA, 1.1 by IVA, or 1.2 4-HA, suggesting that PA acts as an inhibitor with a mixed inhibition type against thrombin, although IVA and 4-HA play inhibitory roles with an uncompetitive inhibition type according to the two decision systems [18].

As shown in Table 2, 1/*K*_m_ increased with increasing 1/*V*_max_ in PA treatment of FXa, although only 1/Vmax increased without altering *K*_m_ in IVA or 4-HA treatment. The constants *K*_cat_ and *K*_cat_/*K*_m_ of FXa decreased in a dose-dependent manner by PA, IVA, or 4-HA treatment, and the *K*_ik_/*K*_iv_ ratio values for FXa inhibition were confirmed to be 1.6 by PA, 5.6 by IVA, or 5.3 by 4-HA. These results indicate that PA has an uncompetitive inhibition type, and IVA and 4-HA exhibit non-competitive inhibition against FXa.

As shown in Table 3, 1/*V*_max_ increased without altering 1/*K*_m_ in PA, IVA, and 4-HA treatment of FXIIIa, and the constants of *K*_cat_ and *K*_cat_/*K*_m_ decreased dose-dependently in the compound-treated groups. The *K*_ik_/*K*_iv_ ratio values for FXIIIa inhibition were found to be 6.1 by PA, 10.4 by IVA, or 6005.6 by 4-HA, indicating that the three compounds have a non-competitive inhibition type against FXIIIa according to the two decision systems.

The IC_50_ and *K*_i_ values of PA were determined to be 1.107 and 1.105 mM for thrombin, 3.087 and 3.073 mM for FXa, and 1.6357 and 1.6349 mM for FXIIIa, respectively. The IC_50_ and *K*_i_ values of IVA were 0.9245 and 0.9228 mM for thrombin, 4.208 and 4.194 mM for FXa, and 1.91649 and 1.91648 mM for FXIIIa, respectively, whereas those of 4-HA were 1.7043 and 1.7025 mM for thrombin, 4.007 and 3.993 mM for FXa, and 1.9566 and 1.9558 mM for FXIIIa, respectively.

### 2.4. Effects of PA, IVA, and 4-HA on Plasma Recalcification

**Figure 5:**
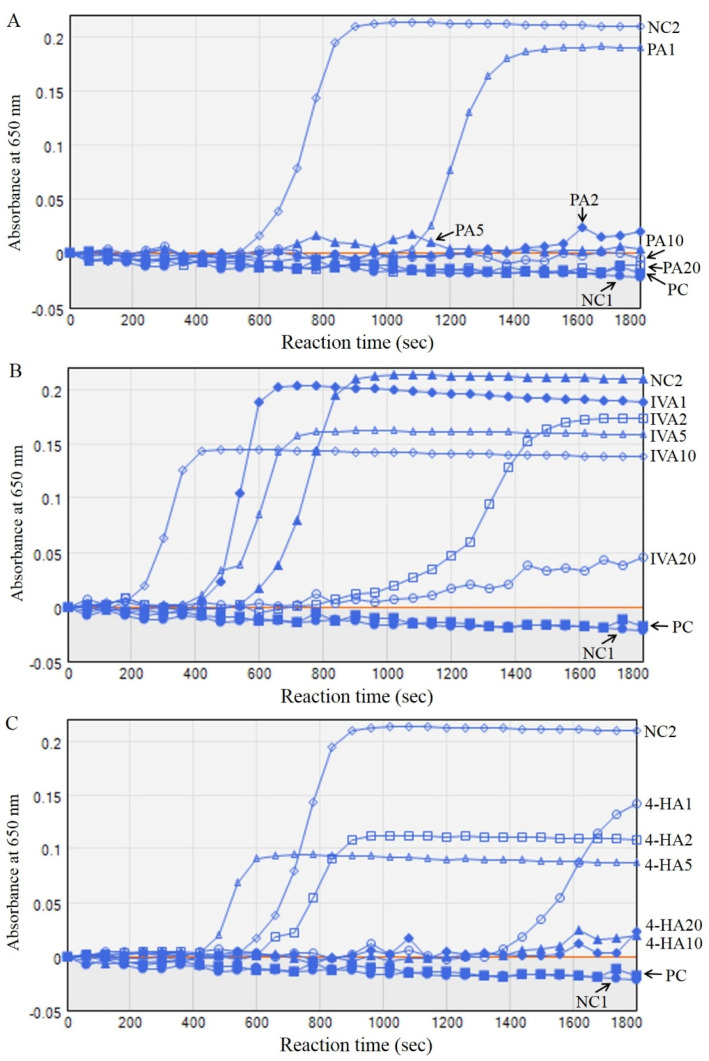
Effect of PA (**A**), IVA (**B**), and 4-HA (**C**) on recalcification time and plasma density. Human platelet-poor plasma was mixed with PA, IVA, and 4-HA dissolved in DMSO in 96-well plates and incubated for 10 min at 37 °C. Thereafter, 100 mM pre-warmed CaCl_2_ was added to the mixture and absorbance was taken every 60 s at 650 nm for 30 min in a microplate reader. The plasma density was presented as the optical density at 650 nm after 30 min. The half time (s) was presented as reaching a half-maximum absorbance value. NC1, plasma only; NC2, plasma treated with CaCl_2_; PC, positive control group (1 U heparin); PA1–20, 1–20 μg of PA treated; IVA1–20, 1–20 μg of IVA treated; 4-HA1–20, 1–20 μg of 4-HA treated.

As shown in Figure 5, the changes in the absorbance of plasma were monitored in each group at 650 nm for 1800 s. Calcium treatment increased the absorbance and density of plasma in the NC2 group compared to the NC1 group without calcium treatment. Although heparin (1U) and PA (5–20 μg) treatment completely delayed the plasma coagulation for 1800 s, IVA and 4-HA treatments partially prolonged the plasma coagulation time. The half-life of plasma coagulation for half-maximum density was 740 ± 20 s, and the final density of plasma after 1800 s was 0.2094 ± 0.0305 of absorbance value at 650 nm in the NC2 group (Table 4). Half-time and plasma turbidity analysis showed that IVA treatment partially promoted the initial response of plasma coagulation by 0.48–0.83-fold, but significantly decreased the final plasma density. In addition, PA and 4-HA treatments resulted in a significant delay in the half-life and plasma coagulation time.

### 2.5. Effects of PA, IVA, and 4-HA on Coagulation Time

**Figure 6:**
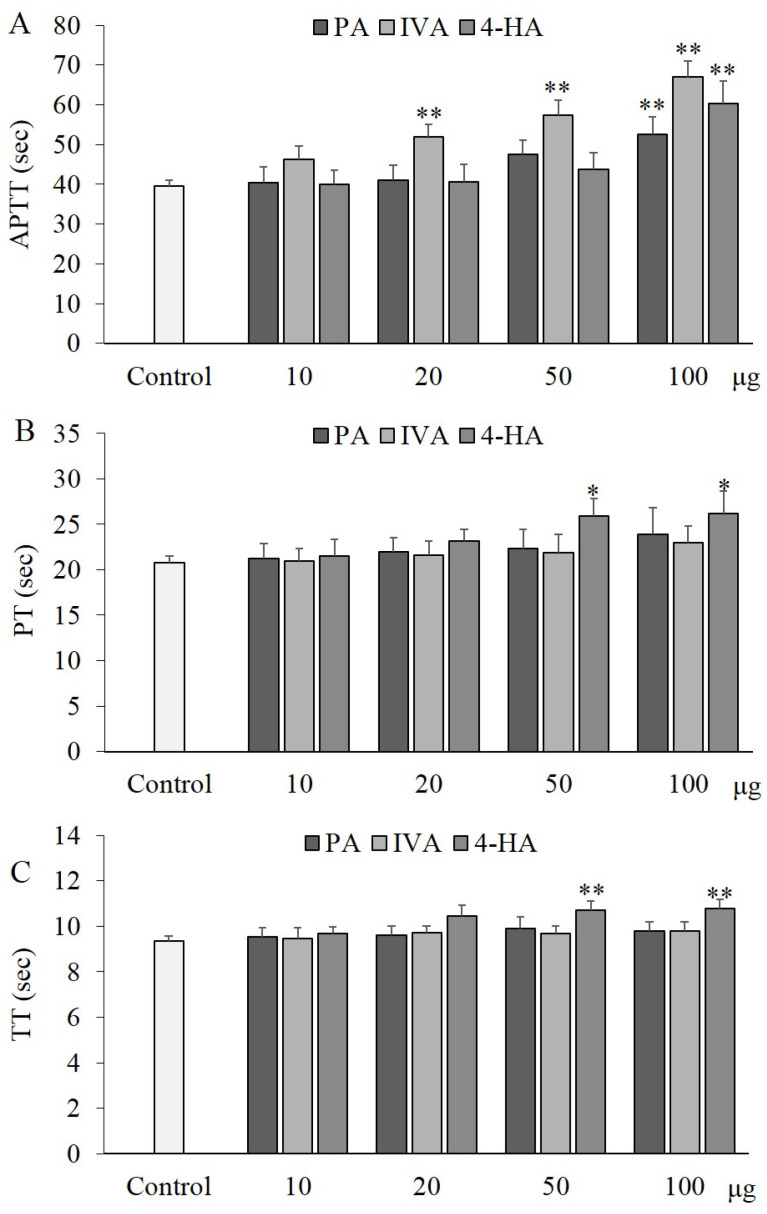
Anticoagulant effect of PA, IVA, and 4-HA. After pretreatment of the compounds with human plasma for 10 min, APTT (**A**), PT (**B**), and TT (**C**) were determined by a coagulometer (Thrombostat 1). Each value is the mean ± SD of triplicate measurements. * *p* < 0.05 and ** *p* < 0.01, compared to each control groups.

As shown in Figure 6A, activated partial thromboplastin time (APTT) was significantly prolonged (*p* < 0.01) by 100 μg of PA (52.5 ± 4.4, 1.33-fold), IVA (67.0 ± 4.0, 1.70-fold), and 4-HA (60.3 ± 5.6, 1.53-fold) treatments, compared to the control group (39.5 ± 1.5 s), and IVA treatment increased APTT in a concentration-dependent manner. Meanwhile, 50–100 μg of 4-HA significantly delayed prothrombin time (PT) and thrombin time (TT) by 25.9–26.2 s (1.25–1.26-fold) (*p* < 0.05) and 10.7–10.8 s (1.14–1.15-fold) (*p* < 0.01), respectively, compared to the control group (20.8 ± 0.7 and 9.4 ± 0.2 s) (Figure 6B). No significant changes were observed in the experimental group treated with 10 or 20 μg of the compound.

### 2.6. Effects of PA, IVA, and 4-HA on Collagen and Epinephrine-Activated Platelet Aggregation

As shown in Table 5, the closure time of blood in the control group was found to be 118.6 ± 2.1 s after treatment with collagen and epinephrine. We found that 100 μg of PA or IVA treatment significantly increased (*p* < 0.05) the closure time by 133.7 ± 5.2 (1.13-fold) or 129.4 ± 3.4 s (1.09-fold), respectively, compared to the control group (118.6 ± 2.1 s). In the 4-HA treatment group, the closure time was prolonged by 134.6 (1.13-fold) and 147.3 s (1.24-fold) at concentrations of 50 and 100 μg, respectively.

### 2.7. Effects of PA, IVA, and 4-HA on Granule Secretion

**Figure 7:**
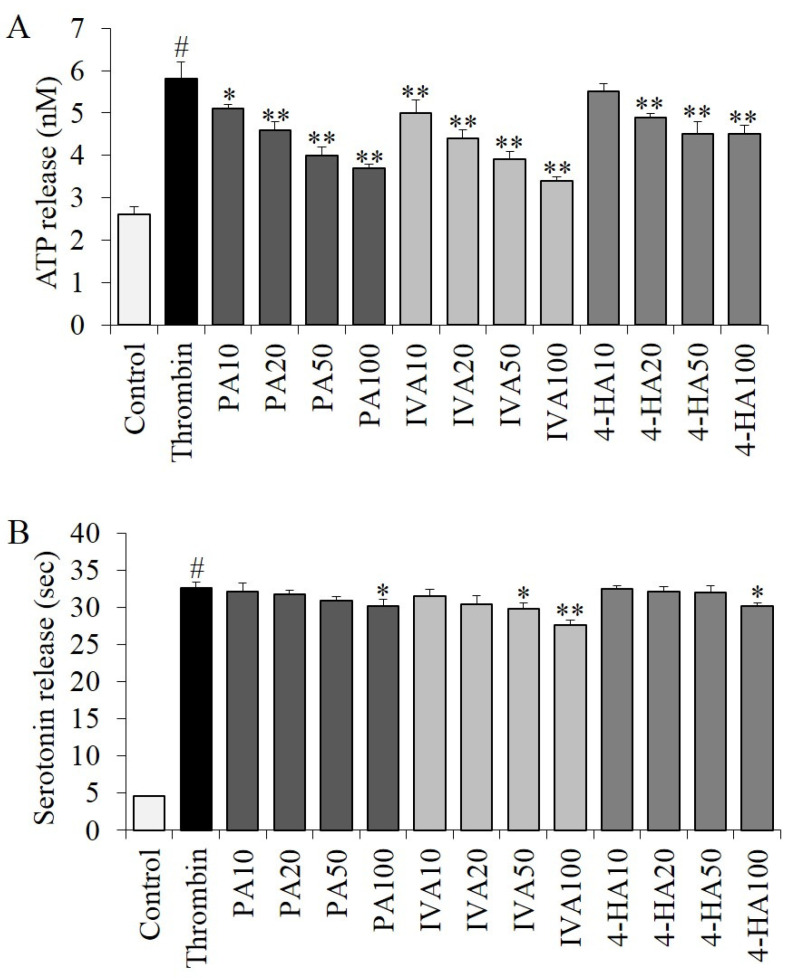
Effect of PA, IVA, and 4-HA on granule secretion. Aliquots of washed platelets pre-treated with or without the compounds for 5 min were stimulated with 0.25 U/mL thrombin at 37 °C for 5 min. After stimulation, the platelets were centrifuged, and the supernatants were collected. Using these supernatants, ATP (**A**) or serotonin (**B**) release was determined according to the manufacturer’s instructions. Each value is the mean ± SD of triplicate measurements. # *p* < 0.01, compared to each control groups. * *p* < 0.05 and ** *p* < 0.01, compared to only thrombin-treated control groups. Thrombin, thrombin treated only; PA10-100, 10–100 μg of PA treated; IVA10-100, 10–100 μg of IVA treated; 4-HA10-100, 10–100 μg of 4-HA treated.

Thrombin treatment significantly stimulated (*p* < 0.01) ATP and serotonin secretion from granules by 2.23-fold (5.8 ± 0.4 nM) and 7.11-fold (32.7 ± 0.7 nM), respectively, compared to the control group (2.6 ± 0.2 and 4.6 ± 0.1 nM) (Figure 7). In the ATP assay (Figure 7A), thrombin-stimulated ATP secretion levels were significantly decreased (*p* < 0.05) by treatment with PA, IVA, and 4-HA compared to the thrombin group without any compound treatment, except the experimental group treated with 10 μg of 4-HA (*p* = 0.915). As shown in Figure 7B, thrombin-stimulated serotonin secretion was inhibited significantly (*p* < 0.05) by treatment with 100 μg of PA, 50–100 μg of IVA, or 100 μg of 4-HA, while no significant changes were observed in the experimental groups treated with low concentrations (10–50 μg) of these compounds.

## 3. Materials and Methods

### 3.1. Materials

Human fibrinogen, thrombin, FXa, FXIIIa, urokinase-type plasminogen activator (u-PA), trizma base, and trizma HCl were purchased from Sigma-Aldrich (St. Louis, MO, USA). Paranitroaniline chromogenic substrates were obtained from Chromogenix (Milan, Italy). Alexa Fluor 488-conjugated fibrinogen was purchased from Invitrogen (Eugene, OR, USA). Other reagents used were of analytical grade and purchased from commercial sources.

### 3.2. Turbidity Assay

Inhibition of fibrin clot formation was determined using a turbidity assay as previously described [10]. The reaction mixtures were prepared by the addition of the compounds, 10 IU u-PA as a positive control (PC), or vehicle as a negative control (NC) 2 in a volume of 100 μL of 5 mM CaCl_2_, 0.5 U/mL of thrombin, and 2.9 mM fibrinogen in 20 mM Tris-HCl (pH 7.4). The mixture was incubated for 5 min at 37 °C, and turbidity was monitored at 405 nm every 1 min for 2000 s using a microplate reader (Molecular Devices, Sunnyvale, CA, USA). The clot density was presented as a percentage of inhibition.

### 3.3. Fibrin Clot Assay

The fibrin clot assay, as previously described [10], was performed to evaluate the improvement in blood circulation by the attenuation of fibrin formation. First, 1.5% fibrinogen, 0.5 U/mL of thrombin, and 5 mM CaCl_2_ in 20 mM Tris-HCl in a volume of 100 μL were added to different concentrations of compounds or 10–20 IU u-PA as PC 1–2. These prepared fibrin clot mixtures were incubated at 37 °C for 30 min. Each tube of the fibrin clot mixture was photographed, and the density of the clots was measured using the ImageJ 1.46 b image analysis software (National Institutes of Health, Bethesda, MD, USA). Inhibitory activity was calculated as follows:

Inhibitory activity (%) = [1 − (density _control without sample_−density _sample_)/density _control without sample_] × 100

Fibrin clots were prepared by mixing 2.6 μM human fibrinogen and 0.4 μM Alexa Fluor 488 fibrinogen with 0.25 U/mL of thrombin in 20 mM Tris-HCl (pH 7.4) in a glass-bottom dish and treated with compounds or 20 IU u-PA as previously described [19]. The mixtures were incubated for 30 min at 37 °C in the dark. Fibrin clots were observed under a fluorescence microscope (Nikon, Eclipse TE 2000-U, Kyoto, Japan) and photographed. Fibrin clots pre-treated with saline (control group) or plasminogen by tissue-type (t-PA) (positive control group). Quantitative analysis of the fluorescent fibrin clots was performed using the ImageJ 1.46 b image analysis software.

### 3.4. In Vitro Antithrombotic Activity

To determine the in vitro antithrombotic activity, a blood clot assay was performed based on a previously described method [7]. All experimental procedures were performed in accordance with the related ethical regulations of Gwangju University, and were approved by the Institutional Animal Care and Use Committee of Jeonnam Institute of Natural Resources Research, Jangheung, South Korea (2021-JINR2001). Fresh mouse blood clots (40 μg) treated with the compounds, 10–20 IU u-PA as PC 1–2, or saline as NC were incubated for 1 h. After incubation, blood clot degradation in each group was analyzed using absorbance values at a wavelength of 410 nm. The maximum absorbance wavelength was determined by measuring the absorption spectra in the wavelength range of 340–850 nm.

### 3.5. Thrombin, FXa, and FXIIIa Assays

To determine the thrombin activity, various concentrations of compounds were treated with 1 U human thrombin in 100 μL of 20 mM Tris-HCl, as previously described [7]. The reaction solutions were incubated for 15 min at 37 °C. After the incubation, 1 mM chromogenic substrate for thrombin (S-2238, HD-Phe-Pip-Arg-*p*-nitroanilide) was added and incubated for 1 h at 37 °C. Absorbance was measured at 405 nm to determine the enzymatic activity. To determine FXa activity, various concentrations of the compounds were mixed with 1 U human FXa in 20 mM Tris-HCl (pH 7.4) in volume of 100 μL, followed by incubation for 15 min at 37 °C. After adding 1 mM *N*-benzoyl-Ile-Glu-Gly-Arg-*p*-nitroanilide, a highly specific chromogenic peptide substrate for FXa, the reaction solutions were incubated for 1 h at 37 °C. The residual activity of FXa was measured at 405 nm and is presented as a percentage of inhibition. To investigate FXIIIa activity, various concentrations of the compounds were mixed with 1 U human FXIIIa in 20 mM Tris-HCl (pH 7.4) in a volume of 100 μL, followed by, incubation for 15 min at 37 °C as previously described [10]. The residual activity of FXIIIa was measured using casein and fibrinogen-HRP, according to the manufacturer’s instructions, and was presented as a percentage of inhibition. The negative control was generated by treatment with only thrombin, FXa, or FXIIIa without treatment with any compound.

### 3.6. Procoagulant Proteases and Fibrinoligase Inhibitor Studies: Kinetic Assays

Kinetic assays were performed using PA, IVA, and 4-HA for procoagulant proteases (thrombin and FXa) and fibrinoligase (FXIIIa). The kinetic constants were calculated using Lineweaver–Burk plots based on the initial reaction rates with different concentrations (0.1, 0.5, 1, and 2 mM) of each substrate for thrombin, FXa, and FXIIIa treated with or without the compounds (0.065, 0.13, 0.33, 0.65, and 1.30 mM [1–20 μg]) in a total volume of 100 μL. The Michaelis–Menten constant (*K*_m_) and maximal velocity (*V*_max_) were calculated from the x and y intercepts, respectively. The catalytic rate constants (*K*_cat_) of each group were derived from the expression *V*_max_/[*E*_o_], where [*E*_o_] = 3.47 μM (thrombin), 27.12 μM (FXa), and 1.60 μM (FXIIIa). The median inhibitory concentration (IC_50_) was defined as the concentration required to reach a 50% reduction in the activity of the three target factors. The *K*_ik_ and *K*_iv_ inhibition constants were calculated using a previously described method [18]. The inhibition type of the compounds against the three target factors was evaluated according to a previously described inhibition type definition [18].

### 3.7. Recalcification Time Assay

Recalcification time was measured in a 96-well plate, according to a previously described method [20], with slight modifications. First, 50 μL of human platelet-poor plasma was mixed with 50 μL of the compounds, vehicle as NC2, or 1 U heparin as PC, and incubated at 37 °C for 10 min. Thereafter, 100 mM pre-warmed CaCl_2_ was added to the mixture and absorbance was measured at 650 nm every 1 min for 30 min using a microplate reader. The plasma density was presented as the absorbance at 650 nm after 30 min. The half time was presented as reaching a half-maximum absorbance value.

### 3.8. Coagulation Assay

A coagulation assay was performed to evaluate the prolongation potential of the compounds in blood coagulation by measuring APTT, PT, and TT, as previously described [20]. After pretreatment of the compounds with human plasma for 10 min, APTT, PT, and TT were determined by a coagulometer (Thrombostat 1) manufactured by Behnk Elektronik, Germany.

### 3.9. Platelet Function Assay

The effects of the compounds on platelet aggregation/activation and adherence in whole blood under conditions of high shear stress flow were evaluated using a collagen/epinephrine cartridge (Dade Behring, Marburg, Germany) and platelet function analyzer (PFA-100), as previously described [20]. Blood samples were drawn from healthy donors using 3.8% (*w*/*v*) sodium citrate. Aliquots of whole blood were treated with different concentrations of compounds and incubated for 5 min at room temperature. Blood samples were added to a collagen/epinephrine cartridge, and the time required for occlusion of the aperture was measured using the analyzer and reported as the closure time. Saline-treated blood was used as a control.

### 3.10. ATP Release Assay

Aliquots of washed platelets (250 × 10^6^/mL) pre-treated with the compounds for 5 min were activated with 0.25 U/mL of thrombin for 5 min at 37 °C, according to a previously described method [20], with slight modifications. After activation, the supernatant from centrifuged platelets was used. ATP release was evaluated using an ATP assay kit (Biomedical Research Service Center, Buffalo, NY, USA), according to the manufacturer’s instructions.

### 3.11. Serotonin Release Assay

Aliquots of washed platelets (250 × 10^6^/mL) pre-treated with the compounds for 5 min were activated with 0.25 U/mL of thrombin in the presence of 1 mM of Ca^2+^ for 5 min at 37 °C, according to a previously described method [20], with slight modifications. After stimulation, the reaction solution was centrifuged at 4 °C and 12,000× *g* for 5 min, and the supernatant was collected for subsequent use. Serotonin release was determined using a serotonin enzyme-linked immunosorbent assay kit (Labor Diagnostika Nord GmbH & Co., Nordhorn, Germany), according to the manufacturer’s instructions.

### 3.12. Hematological Study

Total white blood cells (leukocytes) and red blood cells (erythrocytes) were counted using a hemocytometer, as previously described [20]. Blood was diluted 1:200 with Hayem’s fluid and the cells were counted in a loaded hemocytometer chamber. To count the blood platelets, blood was diluted in a ratio of 1:20 with a diluting fluid, and four large (1 mm^2^) corner squares of the hemocytometer were counted under a light microscope. Aliquots of leukocytes (0.1 mL; 10^3^/mL), erythrocytes (0.1 mL; 10^6^/mL), and platelets (0.1 mL; 10^3^/mL) were treated with or without the compounds for 10 min.

### 3.13. Statistical Analysis

Statistical analysis was performed using the SPSS 17 software (SPSS Inc., Chicago, IL, USA). Data collected and analyzed in this study are expressed as the mean ± standard deviation. Statistical significance in multiple group comparisons was assessed using one-way analysis of variance, followed by a Tukey’s post-hoc test. *P*-values less than 0.05 were considered to be statistically significant.

## 4. Discussion

In response to blood coagulation, circulating platelets and coagulation factors are crucial for the primary process because thrombi are generated by fibrin clotting with fibrinogen (coagulation factor I), thrombin (coagulation factor II), prothrombin (activated coagulation factor X, FXa), fibrinoligase (activated coagulation factor XIII, FXIIIa, or fibrin stabilizing factor), and platelet activation [20,21]. Thrombin is activated by FXa and converts fibrinogen into fibrin, and while polymers and fibrin clots are formed under the effect of FXIIIa. Platelets react to extracellular stimuli and act via interactions between integrin receptors and specific ligands, such as collagen and proteoglycan decorin, on the platelet surface and the binding of adhesive proteins, such as fibrinogen, fibronectin, and Willebrand factor [22]. Ligand-binding induces several intracellular signaling pathways, including fibrin clot retraction, platelet spreading, adhesion stability, and granule secretion [23]. Therefore, strategies to reduce the activity of key coagulation factors, thrombin and FXa, or interfere with their functions and delay the activation of platelets can be used as important tools to suppress excessive blood clot formation and platelet hyperactivation. To investigate whether the inhibition ability of PA, IVA, and 4-HA on key coagulant factors is involved in the formation of fibrin or blood clot, we evaluated the factors activities following compounds treatment. These compounds inhibited the enzymatic activities of procoagulant proteases and fibrinoligase. It has been reported that thrombin and FXa as procoagulant protease, can be controlled by direct or indirect inhibitory pathways [24]. We further investigated the inhibition abilities of three compounds on fibrin or blood clot and platelet activation. We found that PA, IVA, and 4-HA have an ability to alter the composition or structure of fibrin network. The function of coagulation factors and fibrin structure interact with each other, and, in particular, the formation and regulation of the fibrin network affects the reactivity of enzymatic activity of thrombin [24]. In addition, few factors, including the constitution of the fibrin fibers, platelet clot retraction, and the interaction between the thrombin and fibrinogen, affect regulation of fibrin or blood clot. The inhibitory action of PA, IVA, and 4-HA might prevent the formation of fibrin or blood clot via suppression of the interaction between thrombin and fibrinogen, and decrease of platelet activation or thrombin activity.

To date, most thrombin and FXa inhibitors have been produced via chemical synthesis methods and exhibit strong clinical efficacy [25]. Although many studies are focusing on discovering naturally occurring antithrombotic or anticoagulant substances with thrombin and FXa inhibitory properties, these studies are still in their infancy. Thrombin inhibitors with anticoagulant and antithrombotic properties, such as hirudin, heparin, theromin, glycyrrhizin, variengin, and bothrojaracin, are found in various biological groups [26,27,28,29,30,31]. In addition, various chemically synthesized thrombin inhibitors, such as warfarin, bivalirudin, argatroban, melagatran, ximelagatran, and dabigatran, have been studied [32,33,34,35,36,37]. Thrombin inhibitors directly or indirectly target the three domains of the active site and exosites 1 and 2. Exosite 1 serves as the fibrin-binding domain and exosite 2 is the heparin-binding site of thrombin [38]. FXa inhibitors are classified as direct or indirect inhibitors of FXa. Direct FXa inhibitors inhibit FXa without cofactors, whereas indirect FXa inhibitors inhibit FXa via AT-III activity [39]. Oral FXa inhibitors bind in an L-shaped fashion within the FXa active site. The “L” structure includes the S1 and S4 binding sites of factor Xa [40]. At the end of the “L” structure, there is a specific target that can be bound owing to the polar nature of the natural substances, whereas synthetic inhibitors have several aromatic rings and bind via alternative interactions in the S1 and S4 pockets [40]. For the treatment and prevention of cardiovascular diseases, such as atrial fibrillation and acute venous thromboembolism, apixaban, edoxaban, rivaroxaban, and betrixaban have been approved as direct FXa inhibitors, and these agents significantly reduce clot-associated or prothrombinase activity and the risk of stroke or systemic embolism [41,42]. Furthermore, agents known to inhibit platelet activation and aggregation by targeting ADP, cyclooxygenase-1, and glycoprotein IIb/IIIa receptors in cerebral ischemic attack, idiopathic intracranial hypertension, cerebral venous thrombosis, and acute coronary syndromes include ticlopidine, clopidogrel, aspirin, and tirofiban [43,44,45]. Plasmin is formed by the conversion of t-PA or u-PA and hydrolyzes fibrin clots into fibrin degradation products [46]. Streptokinase, tenecteplase, alteplase, and reteplase have been utilized in thrombolysis and clinical studies [47,48,49]. However, most inhibitors of thrombin and FXa, or thrombolytic agents, have side effects, such as hepatotoxicity, renal impairment, thrombocytopenia, osteoporosis, low specificity, decreased platelet or white cell numbers, platelet purpura, and bleeding complications [50,51,52,53]. To overcome these problems, several studies have been conducted on natural sources with anticoagulant properties, depolymerization or degradation, and non-toxic design [54,55,56], In particular, studies on thrombin–FXa dual inhibitors and non-toxic plant food materials are attracting significant attention [25,57]. The present study examines whether the biopolyphenolics PA, IVA, and 4-HA have in vitro anticoagulation activity via measuring recalcification time, APTT, PT, and TT on coagulation system. We found that three compounds strongly prolonged recalcification time and APTT, which evaluates quality of in intrinsic coagulation pathway. It is possible to suggest three compounds exerted in vitro antithrombotic activity via inhibiting the intrinsic pathway through decreases in several coagulation factors, VIII, IX, XI, XII, and vWF [58]. We further investigated the effects of the compounds on the viability of blood cells. We found that these three compounds (up to 50 μg/mL) were not toxic to blood cells. These results provide new information in the toxicity studies of plant-derived biopolyphenolics and insights in biopolyphenolics’s blood compatibility and related studies.

Among phenolic compounds, benzoic acid and its derivatives, such as PA, gallic acid, caffeic acid, ferulic acid, and vanillic acid, are naturally existing compounds found in edible or medicinal plants that have specific structural similarities and carboxylic groups [59]. These substances have strong antioxidant properties and are used in screening studies to assess their functions based on their antioxidant actions for the prevention and treatment of various diseases. PA (3, 4-dihydroxybenzoic acid) is a water-soluble component found in edible vegetables and fruits, and is one of the major benzoic acid derivatives [59]. PA shows various pharmacological effects, including antidiabetic, antioxidant, antibacterial, antitumor, neuroprotective, anti-apoptotic, and anti-inflammatory effects [60,61,62]. Moreover, PA is a major anthocyanin metabolite, which is of great interest in pharmaceutical and nutritional research as it can reach the tissues at concentrations that can elicit various biological effects [61]. PA has protective effects against thrombosis, myocardial fibrosis, and cardiovascular disease by reducing *p*-selectin, GPIIb-IIIa, platelet activation, and by mediating the balance between thromboxane A2 and prostacyclin 2 [13,63,64]. IVA belongs to a class of organic compounds, known as *p*-methoxybenzoic acids. These are benzoic acids, in which the hydrogen atom at position 4 of the benzene ring is replaced by a methoxy group. Meanwhile, 4-HA, a monohydroxybenzoic acid, has a hydroxyl substituent at position 4 of the benzene ring. Although many studies have investigated the protection of cardiovascular functions and improvement of blood circulation by polyphenols [65,66], studies on the improvement effects of IVA and 4-HA, including their antithrombotic, anticoagulation, and anti-platelet effects, are rare. IVA has protective effects on cardiovascular and blood circulation health by mediating the soluble vascular cell adhesion molecule-1 secretion in human umbilical vein endothelial cells stimulated by the tumor necrosis factor-α [65]. Moreover, 4-HA showed IC_50_ values of 300 μM in sodium arachidonate-induced platelet aggregation, and 1–3.4 mM in ADP or collagen-induced platelet aggregation and blood circulation health-protective effects by reducing plasma glucose levels in a dose-dependent manner in diabetic rats [16,17,67].

The present study showed that PA inhibited thrombin, FXa, and FXIIIa via mixed, uncompetitive, and non-competitive inhibition, respectively, whereas IVA and 4-HA inhibited thrombin via uncompetitive inhibition, and FXa or FXIIIa via non-competitive inhibition, suggesting that PA interacts with thrombin or the thrombin–fibrinogen complex to decrease the enzymatic activity of thrombin or to interfere with the thrombin–fibrinogen reaction, and PA acts on FXa, FXIIIa, and their enzyme–substrate complexes to inhibit thrombin activation and fibrin polymer/clot formation. These results indicate that treatment with IVA and 4-HA inhibits the enzymatic activity of thrombin or the thrombin–fibrinogen reaction by binding to the active site (binding or catalytic site) of thrombin or the thrombin–fibrinogen complex and inhibiting the enzyme–substrate interaction of FXa and FXIIIa, thereby inhibiting the activation of the conversion of prothrombin to thrombin and the formation of fibrin-to-fibrin cross-linking polymers. Meanwhile, the function of polyphenolics usually depends on their specific structure and hydroxylation characteristics. Previous studies reported that the presence of a hydroxyl group at C3 provided a crucial role in the inhibition efficacy and additional OH groups in the B-ring may strengthen thrombin or FXa inhibitory action [68,69]. Moreover, quercetin having two OH groups at C3 and C4 in the B-ring generates three hydrogen bonds on active site of S3 pocket in thrombin [68]. Therefore, PA, IVA, and 4-HA, which have hydroxyl groups in C3 and/or C4, may reduce the enzymatic activity by forming hydrogen bonds to the S3 pocket of thrombin [70]. Although the molecular docking of PA, IVA, and 4-HA was not evaluated, it was confirmed that three compounds act as inhibitors of thrombin, FXa, and FXIIIa, respectively, in the kinetics results, and further investigation for molecular docking is needed to understand the inhibitors actions of three compounds on the key coagulation factors.

In conclusion, PA, IVA, and 4-HA have the potential to inhibit fibrin/blood clot formation, coagulation, and platelet function by attenuating fibrin polymer formation or the intrinsic coagulation pathway, decreasing the enzymatic activities of procoagulant proteases or the release of granule constituents, and interrupting the interactions among thrombin, FXa, FXIIIa, and their enzyme–substrate complexes. Our findings reveal the potential mechanisms underlying the in vitro antithrombotic and anticoagulant actions of PA, IVA, and 4-HA, and suggest that these compounds have the potential to serve as inhibitors of thrombin, FXa, and FXIIIa.

## Figures and Tables

**Table 1:** Effect of PA, IVA, and 4-HA on kinetic parameters of thrombin.

Compound (mM)		*K*_m_ (mM)	*V*_max_ (mU/min)	*K*_cat_ (s^−1^)	*K*_cat_/*K*_m_ (mM^−1^s^−1^)	*K*_ik_	*K*_iv_	*K*_ik_/*K*_iv_
Non-treated		0.566 ± 0.025	0.381 ± 0.011	109.87 ± 3.16	196.33 ± 12.61	-	-	-
PA	0.065	0.588 ± 0.017	0.352 ± 0.019	101.67 ± 1.44	172.79 ± 4.83	0.7831	0.0062	125.4
	0.13	0.569 ± 0.011	0.323 ± 0.021	93.29 ± 1.60	163.85 ± 3.19			
	0.325	0.595 ± 0.023	0.295 ± 0.015	85.00 ± 2.13	142.88 ± 3.05			
	0.65	0.516 ± 0.015	0.249 ± 0.009	71.98 ± 1.95	139.46 ± 2.33			
	1.3	0.506 ± 0.021	0.239 ± 0.008	69.08 ± 1.15	136.46 ± 3.35			
IVA	0.065	0.525 ± 0.013	0.304 ± 0.013	87.72 ± 1.32	166.94 ± 2.96	0.0028	0.0026	1.1
	0.13	0.577 ± 0.015	0.326 ± 0.018	94.13 ± 2.08	163.13 ± 3.88			
	0.325	0.410 ± 0.028	0.233 ± 0.010	67.14 ± 0.93	163.92 ± 2.54			
	0.65	0.333 ± 0.026	0.189 ± 0.007	54.42 ± 0.81	163.53 ± 2.07			
	1.3	0.293 ± 0.011	0.167 ± 0.008	48.31 ± 1.05	164.76 ± 3.13			
4-HA	0.065	0.484 ± 0.015	0.349 ± 0.013	100.59 ± 1.41	207.85 ± 4.06	0.0111	0.0092	1.2
	0.13	0.485 ± 0.022	0.341 ± 0.015	98.38 ± 1.24	202.80 ± 3.55			
	0.325	0.449 ± 0.019	0.317 ± 0.016	91.57 ± 0.98	203.81 ± 3.86			
	0.65	0.453 ± 0.020	0.309 ± 0.020	89.25 ± 0.77	197.20 ± 3.15			
	1.3	0.400 ± 0.014	0.273 ± 0.013	78.83 ± 1.01	196.93 ± 2.81			

The maximal velocity (*V*_max_) and the Michaelis–Menten constant (*K*_m_) values were calculated according to Lineweaver–Burk from the data shown in Figure 4. The *K*_ik_/*K*_iv_ ratio was calculated according to Yang et al. [18]. Each value is the mean ± SD of triplicate measurements.

**Table 2:** Effect of PA, IVA, and 4-HA on kinetic parameters of FXa.

Compound (mM)		*K*_m_ (mM)	*V*_max_ (mU/min)	*K*_cat_ (s^−1^)	*K*_cat_/*K*_m_ (mM^−1^s^−1^)	*K*_ik_	*K*_iv_	*K*_ik_/*K*_iv_
Non-treated		2.04 ± 0.09	0.69 ± 0.05	25.27 ± 0.71	12.38 ± 0.45	-	-	-
PA	0.065	1.77 ± 0.05	0.58 ± 0.03	21.36 ± 0.54	12.08 ± 0.48	0.036	0.023	1.6
	0.13	1.59 ± 0.08	0.47 ± 0.02	17.35 ± 0.48	10.93 ± 0.33			
	0.325	1.38 ± 0.04	0.40 ± 0.02	14.67 ± 0.25	10.61 ± 0.29			
	0.65	1.17 ± 0.03	0.33 ± 0.01	12.09 ± 0.29	10.34 ± 0.35			
	1.3	1.17 ± 0.04	0.32 ± 0.01	11.62 ± 0.31	9.94 ± 0.30			
IVA	0.065	2.02 ± 0.07	0.66 ± 0.04	24.20 ± 0.27	12.00 ± 0.41	0.418	0.075	5.6
	0.13	1.99 ± 0.05	0.61 ± 0.02	22.34 ± 0.41	11.23 ± 0.40			
	0.325	2.05 ± 0.05	0.60 ± 0.03	22.23 ± 0.35	10.83 ± 0.31			
	0.65	2.06 ± 0.04	0.56 ± 0.03	20.67 ± 0.32	10.05 ± 0.27			
	1.3	1.92 ± 0.06	0.50 ± 0.04	18.56 ± 0.30	9.69 ± 0.25			
4-HA	0.065	1.90 ± 0.09	0.60 ± 0.03	22.14 ± 0.22	11.65 ± 0.36	0.575	0.109	5.3
	0.13	2.09 ± 0.08	0.59 ± 0.02	21.77 ± 0.26	10.44 ± 0.22			
	0.325	2.17 ± 0.10	0.59 ± 0.04	21.79 ± 0.44	10.03 ± 0.28			
	0.65	2.20 ± 0.09	0.57 ± 0.02	21.18 ± 0.42	9.64 ± 0.21			
	1.3	2.14 ± 0.06	0.55 ± 0.02	20.22 ± 0.37	9.46 ± 0.23			

*V*_max_ and *K*_m_ values were calculated according to Lineweaver–Burk from the data shown in Figure 4. The *K*_ik_/*K*_iv_ ratio was calculated according to Yang et al. [18]. Each value is the mean ± SD of triplicate measurements.

**Table 3:** Effect of PA, IVA, and 4-HA on kinetic parameters of FXIIIa.

Compound (mM)		*K*_m_ (mM)	*V*_max_ (mU/min)	*K*_cat_ (s^−1^)	*K*_cat_/*K*_m_ (mM^−1^s^−1^)	*K*_ik_	*K*_iv_	*K*_ik_/*K*_iv_
Non-treated		0.223 ± 0.010	0.153 ± 0.03	95.3 ± 0.05	427.3 ± 11.7	-	-	-
PA	0.065	0.217 ± 0.011	0.146 ± 0.03	91.1 ± 0.03	420.1 ± 10.2	0.019	0.003	6.1
	0.13	0.220 ± 0.009	0.135 ± 0.02	84.4 ± 0.06	382.9 ± 10.5			
	0.325	0.220 ± 0.007	0.128 ± 0.03	80.1 ± 0.05	364.3 ± 7.1			
	0.65	0.207 ± 0.010	0.115 ± 0.04	71.8 ± 0.07	347.4 ± 6.3			
	1.3	0.205 ± 0.009	0.100 ± 0.02	62.5 ± 0.04	304.6 ± 6.9			
IVA	0.065	0.215 ± 0.012	0.143 ± 0.02	89.3 ± 0.09	415.2 ± 7.5	0.043	0.0041	10.4
	0.13	0.221 ± 0.008	0.135 ± 0.03	84.5 ± 0.07	383.2 ± 6.5			
	0.325	0.214 ± 0.009	0.129 ± 0.04	80.5 ± 0.05	375.3 ± 5.3			
	0.65	0.214 ± 0.006	0.129 ± 0.03	80.5 ± 0.09	375.3 ± 4.9			
	1.3	0.215 ± 0.009	0.110 ± 0.04	68.7 ± 0.08	319.4 ± 4.5			
4-HA	0.065	0.223 ± 0.010	0.142 ± 0.04	88.6 ± 0.10	427.3 ± 6.1	17.875	0.003	6005.6
	0.13	0.223 ± 0.007	0.141 ± 0.04	87.7 ± 0.09	397.3 ± 5.2			
	0.325	0.223 ± 0.011	0.124 ± 0.03	77.2 ± 0.05	346.1 ± 4.6			
	0.65	0.223 ± 0.009	0.113 ± 0.03	70.5 ± 0.07	316.2 ± 7.7			
	1.3	0.223 ± 0.010	0.099 ± 0.04	62.0 ± 0.06	277.7 ± 6.2			

*V*_max_ and *K*_m_ values were calculated according to Lineweaver–Burk from the data shown in Figure 4. The *K*_ik_/*K*_iv_ ratio was calculated according to Yang et al. [18]. Each value is the mean ± SD of triplicate measurements.

**Table 4:** Recalcification time assay of PA, IVA, and 4-HA.

		Half Time at 1/2 Maximum (s)	Fold of Half Time	Absorbance after Reaction
NC1		-	-	0.0224 ± 0.0018
NC2		740 ± 20	1.00	0.2094 ± 0.0305 ^#^
PC		-	-	0.0184 ± 0.0016 **
PA	1	1220 ± 90 *	1.65 ± 0.12 *	0.1898 ± 0.0227
	2–20	-	-	0.0201 ± 0.0012 **(2), 0.0044 ± 0.0009 **(5), −0.0047 ± 0.0004 **(10), −0.0103 ± 0.0011 **(20)
IVA	1	510 ± 25 *	0.69 ± 0.03 *	0.1882 ± 0.0097
	2	-	-	0.1732 ± 0.0106 *
	5	615 ± 20	0.83 ± 0.03	0.1575 ± 0.0052 **
	10	355 ± 30 *	0.48 ± 0.04 *	0.1377 ± 0.0071 **
	20	-	-	0.0462 ± 0.0035 **
4-HA	1	-	-	0.1424 ± 0.0122 **
	2	950 ± 70 *	1.28 ± 0.09 *	0.1086 ± 0.0083 **
	5–20	-	-	0.0866 ± 0.0051 **(5), 0.0233 ± 0.0014 **(10), 0.0205 ± 0.0029 **(20)

Each value is expressed as mean ± SD of at least three independent experiments (one-way ANOVA, post hoc Tukey test. ^#^ *p* < 0.01 versus NC1. * *p* < 0.05, ** *p* < 0.01 versus NC2). NC1, negative control group 1 with only plasma; NC2, negative control group 2 treated with plasma and CaCl_2_; PC, positive control group with plasma and 1 U heparin; PA, experimental group of protocatechuic acid 1–20 μg; IVA, isovanillic acid 1–20 μg; 4-HA, 4-hydroxybezoic acid 1–20 μg.

**Table 5:** PFA-100 assay of PA, IVA, and 4-HA using collagen/epinephrine cartridge.

	Control	PA (μg)				IVA (μg)				4-HA (μg)			
		10	20	50	100	10	20	50	100	10	20	50	100
Closure time (s)	118.6 ± 2.1	119.9 ± 2.4	122.1 ± 1.4	126.3 ± 2.9	133.7 ± 5.2 **	118.5 ± 1.8	119.7 ± 2.8	123.5 ± 2.2	129.4 ± 3.4 *	121.2 ± 1.9	127.7 ± 3.1	134.6 ± 4.5 **	147.3 ± 5.8 **

Each value is expressed as mean ± SD of at least three independent experiments (one-way ANOVA, post hoc Tukey test. * *p* < 0.05, ** *p* < 0.01 versus Control group). PA, experimental group of protocatechuic acid 10–100 μg; IVA, isovanillic acid 10–100 μg; 4-HA, 4-hydroxybezoic acid 10–100 μg.

## Data Availability

Not applicable.

## Sample Availability

Samples of the compounds are available from the author (Choi, J.H.).
